# Unveiling the Burden of Hepatitis A in Salerno, Italy: A Comprehensive 9-Year Retrospective Study (2015–2023) on the Seroprevalence of HAV Antibodies and Age/Sex Distribution

**DOI:** 10.3390/jcm13185534

**Published:** 2024-09-18

**Authors:** Enrica Serretiello, Domenico Iervolino, Giuseppe Di Siervi, Luigi Gallo, Francesca F. Bernardi, Pasquale Pagliano, Giovanni Boccia, Veronica Folliero, Gianluigi Franci, Luca Rinaldi

**Affiliations:** 1 Clinical Pathology and Microbiology Unit, San Giovanni di Dio and Ruggi D’Aragona University Hospital, 84131 Salerno, Italy; enrica.serretiello@unicampania.it (E.S.); giuseppedisiervi200@gmail.com (G.D.S.); gallo.bio@gmail.com (L.G.); 2 Department of Public Health and Infectious Diseases, Sapienza University of Rome, 00185 Rome, Italy; domenico.iervolino608@gmail.com; 3 U.O.D. Tutela Della Salute e il Coordinamento del Sistema Sanitario Regionale—Regione Campania, 80143 Naples, Italy; bernardi.francesca.futura@gmail.com; 4 Department of Medicine, Surgery and Dentistry “Scuola Medica Salernitana”, University of Salerno, 84081 Baronissi, Salerno, Italy; ppagliano@unisa.it (P.P.); gboccia@unisa.it (G.B.); v.folliero@unisa.it (V.F.); 5 UOC Hospital and Epidemiological Hygiene, San Giovanni di Dio and Ruggi D’Aragona University Hospital, 84131 Salerno, Italy; 6 Department of Medicine and Health Sciences “V. Tiberio”, University of Molise, 86100 Campobasso, Italy; luca.rinaldi@unimol.it

**Keywords:** Picornaviridae, hepatitis viruses, pregnancy, vaccines, prophylaxis, chemiluminescence, seroprevalence, COVID-19, SARS-CoV-2 infection, pandemic period

## Abstract

**Background:** Hepatitis A virus (HAV) infection is a significant global cause of viral hepatitis. At present, the anti-HAV vaccine in Italy is proposed exclusively for specific high-risk groups, and a universal vaccination program is not implemented. **Objectives:** This study aimed to assess the level of immunity against HAV in patients of both sexes across age groups ranging from 0 to 95 years admitted to the San Giovanni di Dio e Ruggi d’Aragona Hospital in Salerno, Italy, over a 9-year period (2015–2023). **Methods:** The total HAV seroprevalence by chemiluminescence Vitros system immunodiagnostics (ortho-diagnostics) was obtained by database analysis, stratifying patients for gender and age group in both the pre-pandemic (2015–2019) and pandemic (2020–2023) periods. **Results:** Out of 28,104 samples collected in 2015–2023, 20,613 resulted positive by total HAV immune screening, with a significant reduction in the annualized proportion of events during the pandemic period compared to the pre-pandemic period. HAV was more abundant in males than females in both periods (exceeding the 70%), with a statistically significant decrease in HAV in females in 2015–2019. The 61–70-year-old age group is more susceptible for both genders, with a strong deviation from the 41–50-year-old age group compared to the 51–60-year-old group. The pandemic period affected the number of analyzed samples in 2020. **Conclusions:** The study revealed high HAV seroprevalence, especially in males and individuals aged 61–70 years. There was a notable decrease in seroprevalence during the pandemic compared to pre-pandemic years. These results emphasize the need for ongoing monitoring and suggest that a universal vaccination program could address regional immunity gaps and lower disease incidence.

## 1. Introduction

Hepatitis A virus (HAV) infection is a prominent global contributor to viral hepatitis [1]. It currently leads to approximately 100 million HAV infections and 1.5 million symptomatic cases annually [2,3]. HAV is a positive-sense RNA virus in the Hepatovirus genus of the Picornaviridae family, a nonenveloped small (27 nm) particle in an icosahedral shape, with four genotypes characterized in humans. Compared to hepatitis caused by viruses B and C, HAV cannot cause chronic infections [4]. Initial symptoms are nonspecific, such as nausea, vomiting, fever, malaise, and abdominal pain, followed by bilirubinuria, pale stools, jaundice (2–4 weeks), and occasionally pruritus, which may persist for up to 6 months. The severity and clinical outcome of hepatitis A are highly dependent on age, with approximately 70% of infections in children being asymptomatic, while adults are more likely to show symptoms [4,5,6]. HAV is predominantly transmitted through the fecal–oral rout, with several factors contributing to its spread [4]. Daycare centers are significant sites of transmission, as close contact among children facilitates viral spread, potentially leading to secondary infections in their parents [7]. HAV can be transmitted through contaminated food and water, particularly in settings where food handlers may contaminate food by failing to wash their hands after defecation [8,9]. The virus can also be present on fruits, vegetables, and other raw foods; thus, proper sanitation or cooking is crucial, especially for bivalve mollusks, such as oysters, clams, and mussels, which filter water containing fecal contaminants [10,11]. The epidemiology of HAV infections exhibits significant global variation. HAV is hyperendemic in sub-Saharan Africa and southern Asia, where poor hygiene and sanitary conditions facilitate widespread exposure during early childhood, significantly promoting viral transmission. Regions with intermediate endemicity include the Middle East, Eastern Europe, Latin America, North Africa, and middle-income areas of Asia [12]. In contrast, countries with strong economies, such as the United States and those in Western Europe, have lower HAV infection rates, but exhibit increased susceptibility to the disease among non-immune adults. Notably, HAV remains endemic among Native American tribes in the western United States [13]. In developed countries, key risk factors for HAV transmission include sexual activity between men who have sex with men (MSM), the sharing of contaminated needles, and travel to regions where HAV is endemic [14,15]. Epidemic peaks were observed in Italy in 2017 and across Europe from January to June 2018. Overall, HAV prevalence is higher in developing countries and low-income regions [16]. Vertical transmission of HAV, though rare, has been documented and can lead to complications during pregnancy, such as placental abruption, preterm rupture of membranes, and antepartum hemorrhage [17,18,19]. Additionally, intrauterine transmission of HAV has been associated with neonatal meconium peritonitis [20,21]. Diagnostic methods for confirming HAV infection include serological tests to detect specific IgM and IgG and PCR-based assays to identify HAV RNA [22]. The humoral immune response to HAV structural proteins is initiated before the onset of symptoms. Immunoglobulin M (IgM) antibodies against HAV (IgM anti-HAV) are detectable at or before the onset of clinical illness, typically declining within 3 to 6 months and eventually becoming undetectable by standard diagnostic tests. Immunoglobulin G (IgG) antibodies against HAV (IgG anti-HAV) emerge shortly after IgM, persist for years following infection, and provide lifelong immunity [22,23,24,25]. HAV RNA is detectable within a few days of infection and persists for 3–4 weeks in the blood [26]. The present study aims to highlight the seroprevalence of total HAV in a very large catchment area, such as the San Giovanni di Dio and Ruggi d’Aragona hospitals in Salerno. Examining the HAV distribution by sex and its stratification by age groups make the retrospective analysis complete and useful to monitor the infection variation over time, thus being able to evaluate the data year by year and compare them with the data for pandemic years (2020–2023). It is known that the COVID-19 pandemic has certainly affected non-COVID-19 patient management. All the efforts of the health system were directed toward the fight against the spread of the virus, with several related consequences. Access to hospitals, such as the emergency room, was limited and staying at home was strongly recommended, with a consequent reduction in screening, monitoring of long-term treatments, and new diagnoses, as reported by Galimberti F. et al. [27]. It is essential to study the impact of COVID-19 on HAV seroprevalence by comparing retrospective data with the data obtained during the pandemic.

## 2. Materials and Methods

### 2.1. Sample Processing

Samples were collected from patients aged 0–95 years admitted to San Giovanni di Dio e Ruggi d’Aragona Hospital, Salerno, from January 2015 to December 2023. Samples were collected from several hospital wards: ambulatory, surgery (emergency, maxillofacial, cardiology, and vascular), general and emergency medicine, hematology, dialysis, neurosurgery, neurology, pediatrics, psychiatry, and others. The patients’ serum samples were collected and processed in the Virology laboratory for the in vitro qualitative measurement of human hepatitis A virus antibodies (total anti-HAV) by the manufacturing company’s instructions (ortho-clinical diagnostics, Vitros 3600). A competitive immunoassay technique was used and the amount of bound HRP conjugate was inversely proportional to the concentration of anti-HAV present. A < 0.8 positive sample indicates a reactive sample and the presence of anti-HAV IgG or IgM. Results ≥0.8 and <1.0 indicate a borderline sample. Results ≥1.0 and <4.0 indicate a non-reactive sample, with anti-HAV being negative for IgG or IgM.

### 2.2. Data and Statistical Analyses

All statistical analyses were performed using R software (version 12.1.4), starting from the demographic (age and sex) patients’ data information. To investigate the potential relationships within the data, a chi-squared test was employed to determine whether there was a statistically significant association between sex and HAV positivity, and a probable statistically significant association with the prevalence of HAV and the progression of time.

In addition to the chi-squared test, a Fisher’s exact test was conducted to explore the possible associations between gender and age groups in unevenly distributed data. Furthermore, a z-test was utilized to compare the proportions of HAV-positive cases between males and females and if the difference was significant or merely due to random variations. To identify trends in HAV seroprevalence over time, the Cochran–Armitage trend test was conducted. This test allowed the study to assess whether there was a consistent increase or decrease in HAV cases over the observed period, offering insights into long-term epidemiological trends.

For all statistical tests, the confidence level was set at an alpha value of 5%, ensuring that the results were interpreted with a high degree of statistical rigor. Specifically, the data were interpreted based on the *p*-value outcomes: a *p*-value greater than 0.05 indicated that there was no significant association between the variation in prevalence and time, suggesting that any observed changes could be due to chance. Conversely, a *p*-value less than 0.05 confirmed a statistically significant association.

### 2.3. Limitations

Being a database analysis, considerable information about the patients was not available, such as clinical details, the geographical location of the patient’s origin, and vaccine administration; so, we could not specify if immunity was developed by vaccination or by HAV direct contact. The Vitros “HAV total” test provides the simultaneous immunoassays of IgG and IgM; so, they are not reported and discussed separately in the text.

### 2.4. Ethical Consideration Statement

Ethics approval from the Human Research Ethics Committee was not required for this retrospective study, as it does not contain sensitivity data of the patients, but only the laboratory management data collected from databases, which are not directly associated with the patients.

## 3. Results

### 3.1. Seroprevalence of HAV in the 2015–2023 Timeframe

From 1 January 2015 to 31 December 2023, 28,104 serum samples were processed for HAV total immunoassay at the San Giovanni di Dio and Ruggi d’Aragona Hospital, Salerno, Italy. Of these, 20,613 resulted positive for anti-HAV total antibody screening. The absolute and relative (in brackets) frequencies of positive results on the total HAV screening were reported for the pre-pandemic (2015–2019) and pandemic (2020–2023) periods, as shown in Table 1.

### 3.2. HAV Seroprevalence Positivity in Pre- and Pandemic Periods

To evaluate the possible statistically significant variation over the pre- and pandemic periods and the annualized proportions of positive values over different time periods, a z-test analysis was performed. As reported in Table 2, a *p*-value lower than 0.05 indicates a significant reduction in the annualized proportion of events during the pandemic period compared to the pre-pandemic period.

### 3.3. Distribution of HAV Seroprevalence by Gender and Absolute and Relative Frequencies in the Pre-Pandemic Period (2015–2019)

Among the 10,927 positive patients screened in 2015–2019, the distribution of gender in each year was investigated. A total of 6409 (58.7%) males tested positive for the HAV total immunoassay, with respect to the 4518 positive females (41.3%) (Table 3). From the provided description, it emerges that, for each year, the number of positive males is greater than the female population, implying the greater susceptibility of men to the disease. This statistically significant association between sex and positivity to HAV screening is confirmed by the chi-squared test *p*-value of less than 0.05.

### 3.4. Distribution of HAV Seroprevalence by Gender and Absolute and Relative Frequencies in the Pandemic Period (2020–2023)

In 2020–2023, 57.8% males tested positive in the HAV total immunoassay in comparison to females, with a positive prevalence of 42.2% (Table 4). These data are similar to those of the pre-pandemic period, confirming male exposure to the infection. The male seroprevalence scores, which were higher than females’ scores in the pre-pandemic period, were confirmed also in the pandemic period. Also, in this case, the chi-squared test was performed to determine whether there was a statistically significant association between sex and HAV positivity. A chi-squared test *p*-value of less than 0.05 indicates a correlation among seroprevalence and the male gender.

### 3.5. Distribution of HAV Seroprevalence Divided by Sex per Year with Relative p-Values and p-Value Trends in Pre-Pandemic and Pandemic Eras

**Figure 1:**
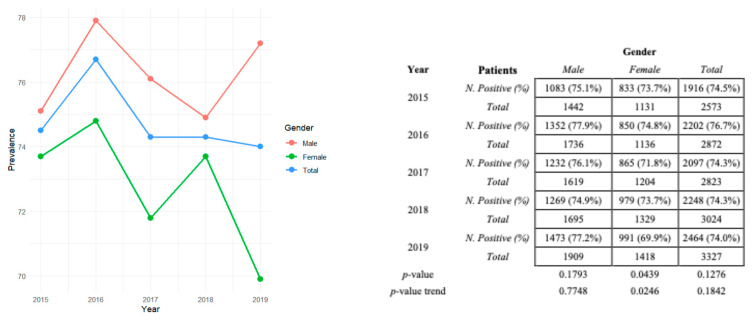

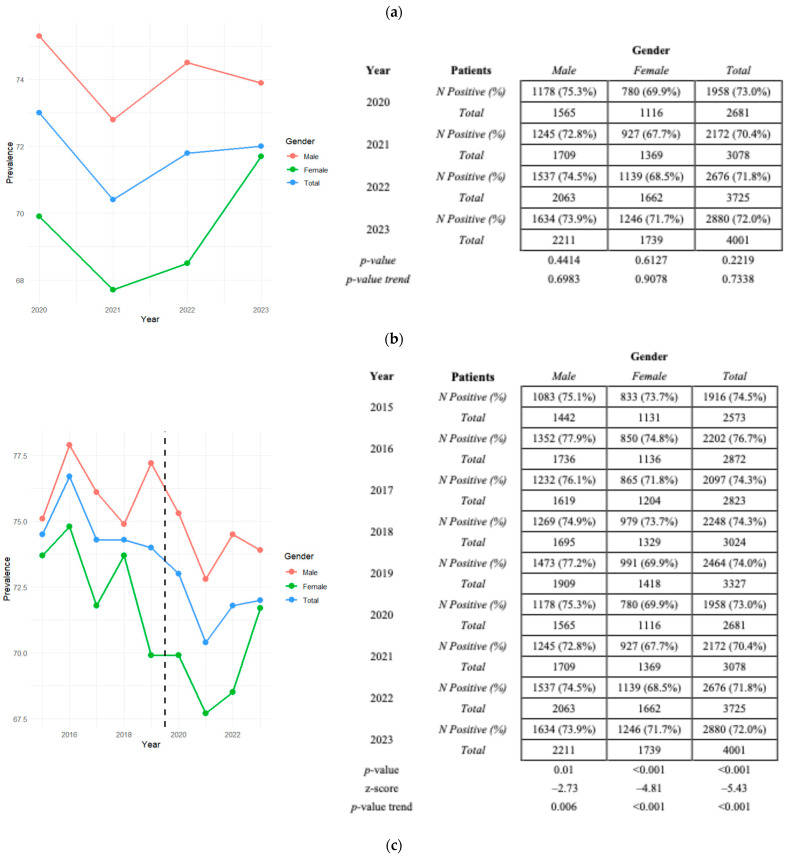
Graphical representation of HAV seropositivity prevalence stratified for the male and female genders and total (male + female + total) in (**a**) 2015–2019, (**b**) 2020–2023, and (**c**) 2015–2023. The relative *p*-value and *p*-value trends in the (**a**) pre-pandemic and (**b**) pandemic periods are also reported. Z-scores in (**c**) represent the trend direction.

We obtained the HAV seroprevalence for different years. No statistically significant variation over time was recorded in the pre-pandemic and pandemic eras. The positivity trend for the male sex showed a very fluctuating trend over time, as the females in the pandemic period, excluding a statistically significant relationship between the variables of time and sex, unlike the pre-pandemic period for females. Furthermore, considering the entire 2015–2023 period, a decreasing positive trend was recorded in all cases analyzed. The trend is observable in the line graph in Figure 1a–c, beyond the absolute and relative values of the positive distribution of males and females in each year analyzed. As detectable from the *p*-value and *p*-value trend reported in Figure 1a,b, statistically significant decrease in HAV in females in 2015–2019 is clear, but not in 2020–2023. The “*p*-value” reported in the tables indicates whether there is a statistically significant association between time and positivity to the disease (chi-squared test); the “*p*-value trend” refers to the Cochran–Armitage trend used to identify trends in HAV prevalence over time, and the z-score value (Figure 1c) informs us about the trend’s direction over the 9 years investigated.

### 3.6. Distribution of HAV-Positive Patients Stratified by Age Group

Total patients positive for HAV immunoassays in the pre-pandemic and pandemic periods were stratified by age group. In particular, the highest percentage of HAV seroprevalence positivity is observed for the 61–70-year-old age group in the pre-pandemic period and 61–70- and 71–80-year-old age groups in the pandemic period, indicating a higher susceptibility among older individuals to become immunized.

### 3.7. Distribution of HAV Seroprevalence by Gender/Age Group in Both the Pre-Pandemic and Pandemic Periods

A stratification of the total positive samples by age group and by gender was performed. As reported in Table 5, the age groups investigated were individuals aged 0–10, 11–20, 21–20, 31–40, 41–50, 51–60, 61–70, 81–90, and 90–95 years. Although the number of total positives may fluctuate over the years, the age distribution of positive cases seems to remain constant from year to year, with a large increase from the 41–50-year-old class to the 51–60-year-old class. Furthermore, a decrease is recorded, probably due to the lack of survival of the patients in the transition to the 81–90-year-old class.

A significant variation in HAV positivity for the male and female age groups in almost all years emerged from the data, confirming the previously considered hypothesis that males aged 61–70 years are susceptible to the infection, in comparison to female positivity, with a percentage around 30. A Fisher’s exact test was used to determine whether there is a significant difference in the frequency distribution between males and females in different age groups. In the pre-pandemic period, female seroprevalence was higher than the male ratio in the 51–60- and 81–90-year-old age groups, the latest also being confirmed in the pandemic period.

## 4. Discussion

The present work aims to assess the level of immunity against hepatitis A in patients’ samples collected in the San Giovanni di Dio e Ruggi d’Aragona hospital, which has a high number of patients from Salerno, Campania. The main purpose was to investigate the positivity rate in the total analyzed samples over the nine years to evaluate the variations (regressions or increases) in HAV immunity diffusion. The positivity prevalence of HAV in the 2015–2023 period exceeds 70% of annual positivity results, even if in 2020 and 2021 a lower number of positives on a smaller number of processed samples was recorded. Table 1 shows the values of positive and negative cases in the 2015–2023 timeframe. The data indicate a reduced HAV seroprevalence in the pandemic period.

In 2023, Italy reported 267 cases of hepatitis A, according to the Integrated Epidemiological System of Acute Viral Hepatitis (SEIEVA), coordinated by the Istituto Superiore di Sanità. This represents an increase from the previous year. The regions most affected were Lombardy (55 cases), Tuscany (43), Emilia-Romagna (29), Marche (28), and Lazio (27). Males were more frequently affected, constituting 59% of the cases, and there was a slight rise in pediatric cases compared to the previous year (45 vs. 39). The most affected age groups were 35–54 years (25.1%) and 25–34 years (19.1%). Three deaths were reported among women aged 77 to 80 years, including cases of hepatorenal syndrome and fulminant hepatitis A with encephalopathy. Major risk factors included the consumption of raw or undercooked shellfish (35.5%), travel to endemic areas (31.9%), and sexual intercourse between men (24.6%). The findings highlight the need for increased vaccination awareness among high-risk groups, including sexually active homosexuals, travelers to endemic regions, and individuals with chronic liver disease. Notably, there was a peak in positivity in 2017, with the 25–34-year-old age group being the most affected [28,29]. In 2021, 30 EU/EEA countries reported a total of 3864 hepatitis A cases, marking a 65.7% decrease from 2019 and a 12.3% decrease from 2020. Males were more frequently affected than females, with the highest prevalence observed in the 5–14-year-old age group. Data on HAV sub-genotypes were provided by four countries—Iceland, Ireland, Norway, and Sweden—for 96 cases (2.5% of the total). Among these, 46 cases were of sub-genotype IA, 43 sub-genotype IB, and 7 sub-genotype IIIA [30]. As reported and discussed below, our investigation of HAV seroprevalence also assessed the gender most exposed and the age group most susceptive to HAV counteraction.

Table 2 shows a decrease in seroprevalence during the pandemic period compared to the pre-pandemic period. This reduction can be attributed to various containment measures implemented at the onset of the pandemic, including travel restrictions, reduced social interactions, limitations on dining out, and decreased healthcare utilization for mild symptoms. In Bulgaria, there was a notable increase in HAV prevalence among hospitalized patients in May 2020 and April 2021 compared to the previous two years [31]. This increase coincides with enhanced the SARS-CoV-2 surveillance of patients with liver-related symptoms, reflecting the hepatic tropism shared by both SARS-CoV-2 and HAV [32]. The literature suggests that the severity of COVID-19 may be associated with an increased susceptibility to HAV [33]. Unlike hepatitis C (HCV), hepatitis B (HBV), and hepatitis D (HDV), which can lead to chronic infection and liver fibrosis, HAV does not cause chronic conditions, but may rarely result in fulminant hepatitis and acute liver failure [34,35]. Additionally, the impact of COVID-19 on HAV epidemiology is compounded by the effects of war, which influences migration patterns and sanitary conditions, including access to clean water [36]. An increase in the incidence of all three types of viral hepatitis was recorded in Ukraine during the period of 2018–2023, analyzing the impact of the COVID-19 pandemic and war on infection and disease spread [37].

Subsequently, we checked whether immunity for hepatitis A was more representative among males or females, in accordance with the SEIEVA data reported [29]. Throughout the 9-year study period, males consistently showed a greater susceptibility to HAV infection than females (Table 3 and Table 4). Although there was a notable decrease in the number of positive cases among males in 2020, this was attributed to a reduced total number of healthcare visits; however, the higher prevalence among males persisted. The statistical analysis revealed a significant association between the male sex and increased likelihood of HAV infection (*p* < 0.05), with male seroprevalence remaining higher than female seroprevalence in both the pre-pandemic and pandemic periods. The trend analysis of seroprevalence over time indicated a statistically significant decline in positivity among females during the 5 years preceding the pandemic. In contrast, male positivity showed a considerable fluctuation without a significant time-related trend. This pattern persisted during the pandemic, with both male and female positivity showing varying trends over time, and no statistically significant relationship was found between time and sex (Figure 1a).

From 2015 to 2023, the absolute number of positive cases increased for both men and women. However, the proportion of positive cases relative to negative cases decreased over time. For men, the absolute number of positive cases increased and the rate of increase in negative cases exceeded that of positive cases, resulting in a decrease in the proportion of positive cases (Figure 1c). The very low *p*-value supports the statistical significance of this decreasing trend. Statistical analysis confirms a statistically significant trend in the decreasing proportion of positive cases relative to negative cases from 2015 to 2023. Similarly, for women, both the numbers of positive and negative cases increased, and that the proportion of positive cases relative to negative cases decreased over time. A statistically significant trend in this decreasing proportion occurred, with negative cases increasing more rapidly than positive cases, resulting in a reduced ratio of positive to negative cases. Overall, despite an increase in the total number of positive cases, the proportion of positive cases to negative cases decreased over time. The statistical data reveal a significant downward trend in this proportion, indicating that negative cases are growing at a faster rate than positive cases, which is statistically significant (Figure 1a–c).

**Figure 2:**
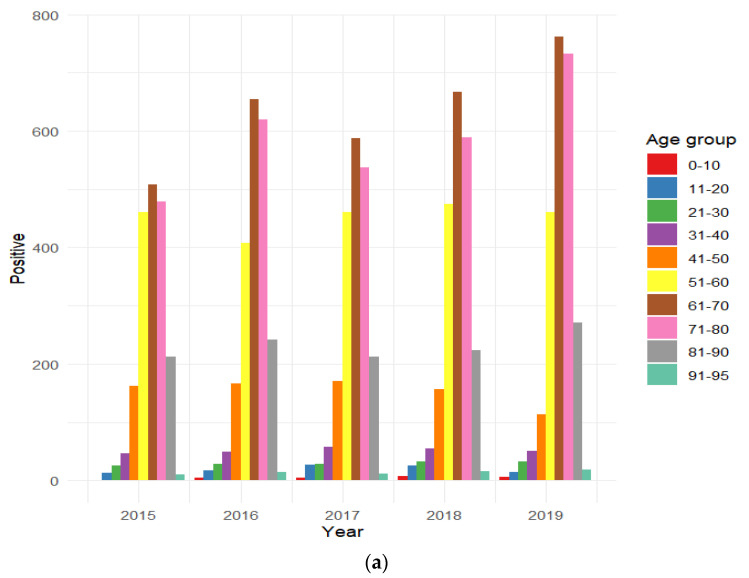

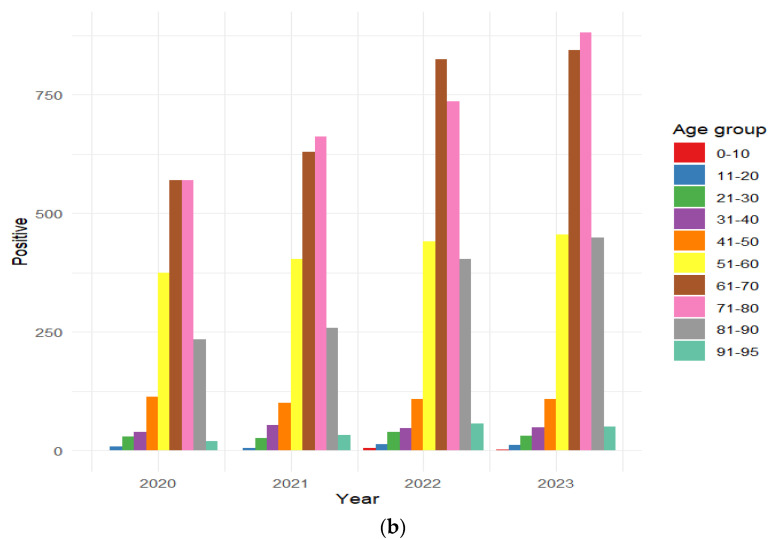
Distribution of HAV-positive individuals divided by age group for each year in the (**a**) pre-pandemic and (**b**) pandemic eras.

The stratification of positive cases by age reveals that the age distribution of HAV infections remains relatively stable over the years, with the 61–70-year-old age group being the most affected in both periods (Figure 2a,b). Seroprevalence increases notably from around the age of 40 years and is less common in younger age groups. These findings align with a study from Chile, which also observed lower positivity rates in children and higher susceptibility in older populations, leading to sporadic outbreaks [38]. The low prevalence of HAV in childhood may be attributed to the challenge of detecting mild or asymptomatic cases, leading to potential underestimations [39]. An analysis of individuals aged 1 to 18 years in Florence, Tuscany, revealed that while HAV endemicity is low in the area, seroprevalence is notably higher among foreign children and adolescents [40]. Sagnelli et al. studied HAV seroprevalence in 2830 patients with chronic liver disease and found that females had a slightly higher exposure rate than males. The study also observed that HAV positivity increased with age, which is consistent with our findings [41]. As noted by Ansaldi et al. in 2006, age is a key predictor of HAV immunity, with higher positivity rates observed in older cohorts who acquired the infection in childhood. This underscores the significance of scientific discoveries and their role in protecting younger populations [42]. Increased awareness and vaccination efforts have played a key role in controlling hepatitis A virus (HAV) transmission. The SEIEVA system reports a slight rise in HAV incidence from 2021 to 2020, but a general decrease from the 2017–2018 peak. Our findings indicate higher seroprevalence in men and a peak in the 60–70-year-old age group, contrasting with the SEIEVA data that show a balanced male to female ratio and a peak in the 50–60-year-old age group. Effective vaccination, especially for travelers and those in contact with infected individuals, and heightened parental awareness about diet and hygiene have contributed to lower exposure rates. Ongoing monitoring is crucial to prevent outbreaks, particularly in high-risk environments and among immunocompromised individuals, as asymptomatic children can facilitate the spread of HAV [43]. Universal childhood vaccination in countries of intermediate endemicity is strongly recommended by the WHO [44].

HAV seroprevalence papers in Italy are quite dated, and most of them concern a small cohort of patients (particularly children and young people, or are organized by demographic area, such as North, Central, and Southern Italy) [45,46]. Therefore, an update on the spread of HAV in Southern Italy is urgent. During the pandemic, non-urgent medical procedures, hospitalizations, and routine check-ups were postponed or canceled. Many clinics suspended operations, and patients avoided hospitals due to a fear of COVID-19, leading to the reduced monitoring of chronic conditions and management of acute issues. Additionally, alternative therapies, such as ozone therapy, were explored for managing COVID-19 positive patients [47]. In Italy, there was a 10% to 33% decrease in the use of hospital emergency services during the pandemic, leading to a concerning 43% increase in out-of-hospital mortality during the lockdown [4]. The study on HAV seroprevalence in Salerno confirmed that the observed decline in cases in 2020–2021 was due to fewer samples being analyzed. Enhanced hygiene practices and other pandemic-related measures likely contributed to a reduced prevalence of enteric viruses, including HAV, as supported by studies showing a correlation with improved handwashing and food safety [48]. Investigating the impact of the COVID-19 pandemic on hepatitis A virus (HAV) vaccination rates among children is essential. Effective strategies for early detection, epidemiological surveillance, and vaccination are crucial for controlling infections and reducing unnecessary antibiotic use. Targeted diagnostic analyses are necessary to prevent antibiotic resistance. Monitoring HAV genotypes is also important for identifying local variants and detecting potential outbreaks, including cross-border ones. The emergence of rare HAV strains, as noted by Ramachandran et al., emphasizes the need to track changes in HAV molecular epidemiology [49].

## 5. Conclusions

Total HAV seroprevalence in Salerno is notably high, exceeding 70%. The study identified elevated seroprevalence rates, particularly among males and individuals aged 61–70 years. However, the COVID-19 pandemic significantly affected HAV screening, prevention, and management strategies. A marked decline in seroprevalence was observed during the pandemic compared to the pre-pandemic period. It is crucial to emphasize that the reduced positivity rates recorded during the pandemic do not reflect a true decline in HAV immunity. Instead, this decrease is attributed to the reduced number of individuals seeking healthcare services due to the pandemic’s impact. Continuous monitoring and implementation of containment strategies are essential to promote the spread of HAV. Regular surveillance and timely interventions can contribute significantly to reducing HAV transmission and safeguarding public health in the region.

## Figures and Tables

**Table 1:** Positive, negative, and total samples analyzed for each of the nine years investigated, reported as a number and percentage (in brackets).

Years	2015	2016	2017	2018	2019	2020	2021	2022	2023	2015–2023
Samples										
Positive n. (%)	1916 (74.5)	2202 (76.7)	2097 (74.3)	2248 (74.3)	2464 (74.1)	1958 (73)	2172 (70.4)	2676 (71.8)	2880 (72)	20,613 (73.3)
Negative n. (%)	657 (25.5)	670 (23.3)	726 (25.7)	776 (25.7)	863 (25.9)	723 (27)	906 (29.6)	1049 (28.2)	1121 (28)	7491 (26.7)
Total samples n.	2573	2872	2823	3024	3327	2681	3078	3725	4001	28,104

**Table 2:** Absolute and relative values for HAV-positive (male plus female), HAV-negative (male plus female), total HAV (positive + negative), and relative *p*-value (z-test). Abbreviations: M = Male; F = Female.

	Positive Absolute and Relative Frequences	Negative Absolute and Relative Frequences	Total	*p*-Value
HAV (2015–2019) N. (%)	10,927 (74.7%)	3692 (25.3%)	14,619	0.009
HAV (2020–2023) N. (%)	9686 (71.8%)	3799 (28.2%)	13,485	

**Table 3:** Total seroprevalence distribution of female and male patients positive for HAV with respect to the total positive amount for each year is reported as a number and percentage (in brackets) during the pre-pandemic period (2015–2019).

Years Gender	2015	2016	2017	2018	2019	2015–2019	Positive and Negative	Positive N. (%)
**Female** N. (%)	833 (43.5)	850 (38.6)	865 (41.2)	979 (43.5)	991 (40.2)	4518 (41.3)	n = 6218	4518 (72.7)
**Male** N. (%)	1083 (56.5)	1352 (61.4)	1232 (58.5)	1269 (56.5)	1473 (59.8)	6409 (58.7)	n = 8401	6409 (76.3)
**Total positive** N	1916	2202	2097	2248	2464	10,927	*p* **-value**	<0.001

**Table 4:** Total seroprevalence distribution of female and male patients positive for HAV with respect to the total positive amount for each year is reported as a number and percentage (in brackets) during the pandemic period (2020–2023).

Years Gender	2020	2021	2022	2023	2020–2023	Positive and Negative	Positive N. (%)
Female N. (%)	780 (39.8)	927 (42.7)	1139 (42.6)	1246 (43.3)	4092 (42.2)	n = 5940	4092 (68.9)
Male N. (%)	1178 (60.2)	1245 (57.3)	1537 (57.4)	1634 (56.7)	5594 (57.8)	n = 7545	5594 (74.1)
Total positive N.	1958	2172	2676	2880	9686	*p*-value	<0.001

**Table 5:** Prevalence of males and females per year divided by age groups and relative *p*-values in the (a) pre-pandemic and (b) pandemic eras.

(a)	2015		2016		2017		2018		2019		2015–2019	
Age/Gender	M	F	M	F	M	F	M	F	M	F	M	F
**0–10**	0%	0.1%	0.1%	0.4%	0.2%	0.3%	0.5%	0.1%	0.2%	0.3%	0.2%	0.2%
**11–20**	0.7%	0.6%	1.0%	0.4%	1.8%	0.6%	1.4%	0.8%	0.8%	0.3%	1.2%	0.5%
**21–30**	1.8%	0.6%	1.0%	1.6%	1.0%	1.8%	1.6%	1.3%	1.7%	0.8%	1.4%	1.2%
**31–40**	2.7%	2.0%	2.4%	2.0%	3.2%	2.1%	2.2%	2.8%	2.6%	1.2%	2.6%	2.0%
**41–50**	8.4%	8.5%	7.6%	7.5%	8.1%	8.1%	6.9%	7.0%	4.4%	5.0%	7.0%	7.1%
**51–60**	22.3%	26.3%	18.4%	18.6%	21.1%	23.1%	20.4%	22.0%	17.7%	28.3%	19.8%	22.0%
**61–70**	26.8%	26.2%	30.2%	29.0%	29.5%	26.0%	31.0%	28.0%	32.4%	28.8%	30.1%	27.6%
**71–80**	27.0%	22.3%	29.2%	26.4%	25%	26.5%	26.5%	25.8%	29.6%	30.0%	27.6%	26.3%
**81–90**	9.8%	12.7%	9.7%	13.0%	9.6%	10.9%	9.3%	10.8%	10.2%	12.2%	9.7%	11.9%
**90–95**	0.5%	0.6%	0.4%	1.0%	0.6%	0.6%	0.3%	1.2%	0.4%	1.2%	0.4%	1.0%
Total	56.5%	43.5%	61.4%	38.6%	58.8%	41.2%	56.5%	43.5%	59.8%	40.2%	58.7%	41.3%
Total positive	1916		2202		2097		2248		2464		10,927	
*p*-value	0.022		0.045		0.059		0.087		0.002		<0.001	
**(b)**	**2020**		**2021**		**2022**		**2023**		**2020–2023**			
**Age/Gender**	**M**	**F**	**M**	**F**	**M**	**F**	**M**	**F**	**M**	**F**		
**0–10**	0%	0%	0%	0%	0.2%	0.3%	0.1%	0%	0.1%	0.1%		
**11–20**	0.2%	0.8%	0.1%	0.4%	0.2%	1.0%	0.3%	0.5%	0.2%	0.7%		
**21–30**	1.5%	1.5%	1.5%	0.9%	1.5%	1.4%	1.5%	0.5%	1.5%	1.0%		
**31–40**	2.3%	1.5%	2.8%	1.9%	1.4%	2.4%	1.7%	1.7%	2.0%	1.9%		
**41–50**	6.3%	5.1%	4.7%	4.6%	3.4%	4.8%	3.2%	4.4%	4.3%	4.7%		
**51–60**	20.0%	17.8%	18.6%	18.7%	16.7%	16.0%	16.3%	15.2%	17.7%	16.7%		
**61–70**	29.6%	28.2%	31.3%	25.9%	33.7%	27.0%	30.7%	27.4%	31.4%	27.1%		
**71–80**	28.4%	30.3%	30.5%	30.4%	28.6%	26.0%	32.3%	28.3%	30.1%	28.5%		
**81–90**	11.4%	12.8%	9.8%	14.7%	12.8%	18.1%	12.9%	19.1%	11.9%	16.6%		
**90–95**	0.3%	1.9%	0.7%	2.5%	1.5%	3.0%	0.9%	2.9%	0.9%	2.6%		
Total	60.2%	39.8%	57.3%	42.7%	57.4%	42.6%	56.7%	43.3%	57.8%	42.2%		
Total positive	1958		2172		2676		2880		9686			
*p*-value	0.006		<0.001		<0.001		<0.001		<0.001			

## Data Availability

The original contributions presented in the study are included in the article, further inquiries can be directed to the corresponding authors.
